# Efficacy of Individual Placement and Support (IPS) on Employment, Education, and Training in Young Adults With Early Psychosis—A Randomized Controlled Trial

**DOI:** 10.1002/brb3.70469

**Published:** 2025-05-05

**Authors:** Dorothea Jäckel, Andreas Bechdolf, Eva Burkhardt, Michèle Kallenbach, Marie‐Luise Gamig, Anna Mößnang, Karolina Leopold

**Affiliations:** ^1^ Vivantes Klinikum Am Urban, Department of Psychiatry, Psychotherapy, and Psychosomatics Incorporating FRITZ at Urban and Soulspace Vivantes Urban Hospital and Vivantes Friedrichshain Hospital Berlin Germany; ^2^ Charité–Universitätsmedizin Berlin, Department of Psychiatry and Psychotherapy CCM corporate member of Freie Universität Berlin and Humboldt‐Universität zu Berlin Berlin Germany; ^3^ German Center for Mental Health (DZPG), partner site Berlin Potsdam Berlin Germany; ^4^ University Hospital for Child and Adolescent Psychiatry and Psychotherapy, University of Bern Bern Switzerland; ^5^ Klinik für Psychiatrie und Psychotherapie Universität Carl Gustav Carus Dresden Germany

**Keywords:** early intervention, early psychosis, functional recovery, NEET, supported education, supported employment, training

## Abstract

**Objective:**

To explore the efficiency of IPS on employment, education, and training (EET) outcomes in young adults with early psychosis.

**Methods:**

Monocenter parallel arm, randomized controlled trial.

**Intervention:**

Individual placement and support (IPS) according to the IPS‐Y Fidelity Scale with an add‐on of up to eight sessions of adherence therapy.

**Main outcome measures:**

EET at least 15 h per week for at least 1 week in the follow‐up. Secondary outcomes included EET rates, duration in EET, and total wages. Additionally, subsample analyses were carried out.

**Results:**

A total of 94 young adults (18–35 years) with early psychosis in an outpatient psychiatric service were randomly assigned to 12‐month IPS according to program fidelity and standard care versus standard care alone. Four patients were excluded from the analysis because of early dropout after baseline, leaving 90 participants—46 in the IPS group and 44 in the TAU group—for intention to treat (ITT) analysis. EET rate for at least 15 h per week was significantly higher in the IPS group, 78% (36/46) versus 55% (24/44) in the TAU group. The percentage of participants in EET for at least 1 week in the follow‐up was 83% (38/46) versus 59% (26/44). The number of weeks in EET was significantly higher in the IPS group (mean = 31.5 weeks, SD = 20.5 versus mean = 18.4 weeks, SD = 21.2), and the total wages were higher in favor of the IPS group (mean = €10,242, SD = 13,437 versus mean = €5217, SE = 7871).

**Conclusion:**

IPS integrated into outpatient psychiatric services improves EET in young adults with early psychosis in Germany.

## Graphical abstract

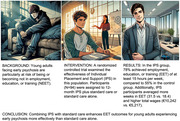

## 1 Introduction

The majority of mental disorders occur in adolescence and the early stages of adulthood, with the peak age of onset at 14.5 years and around 63% of individuals having experienced the onset before the age of 25 (Solmi et al. 2022). Young adults with mental disorders have a twofold higher risk of dropping out of higher education and vocational training and becoming unemployed, that is, “Not in Employment, Education, or Training” (NEET), than people without mental disorders (Hofstra et al. 2023). The prevalence rate of NEET status for individuals with mental health conditions is high, from 30% to 54% (Gariépy et al. 2022; Maraj et al. 2019; Lindhardt et al. 2022; Griffiths et al. 2018). Young adults with early psychosis (EP) are more vulnerable to NEET, face career barriers, and have a higher risk of poverty because they are less educated than their peers.

Prior studies have shown that competitive employment and professional education are among the most essential treatment goals for people with EP (Ramsay et al. 2011;; Secker and Gelling 2006 de Waal et al. 2018). Still, access to effective vocational interventions remains limited in this population (Bond and Drake 2014). Early vocational and educational interventions for young adults with EP can help achieve adequate social roles, meet development needs, and prevent social expenditures and poverty (Bond and Drake 2014).

The current most effective method to address unemployment and enhance functional recovery, particularly competitive employment, education, and training, is Individual Placement and Support (IPS) (Bond et al. 2023). IPS is an evidence‐based method to supported employment (SE) and supported education (SEd) for young adults with severe mental illness (SMI). IPS supports young adults in choosing, getting, and maintaining competitive employment or education/training according to the IPS principles and program fidelity (Drake et al. 2012). Evidence for the effectiveness of IPS has been shown to be superior to other vocational interventions in more than 30 RCTs to date (Bond and Drake 2014; Kinoshita et al. 2013; Marshall et al. 2014; Modini et al. 2016; Wallstroem et al. 2021; Frederick and VanderWeele 2019; de Winter et al. 2022; Brinchmann et al. 2020), for young adults under 30 years of age with SMI, as well as for adults aged 18–65 with SMI (Bond et al. 2023;; Bond et al. 2016). The effects of IPS on education and training for young adults and people with EP are less clear (Bond et al. 2023). These findings demonstrate the need for more rigorous studies based on IPS fidelity criteria examining EET (Bond et al. 2023 Bond et al. 2015; Aguey‐Zinsou et al. 2022). To date, there are eight international RCTs exploring IPS for young adults with mental health conditions (Bond et al. 2023; van Duin et al. 2021; Erickson et al. 2020), of which two studies combined IPS with additional interventions to enhance IPS's effectiveness (van Duin et al. 2021;Nuechterlein et al. 2020). Until now, no RCT on IPS for young adults with EP existed in German‐speaking countries.

### 1.1 Aims of the Study

The study aimed to investigate the efficiency of IPS in addition to standard care in outpatient psychiatric services compared with treatment as usual (TAU) vocational and educational outcomes for young adults with EP (Table 1). The primary outcome criterion was the percentage in EET for at least 15 h per week and for at least 1 week during the 12‐month follow‐up period. The secondary outcomes included further EET measures for the whole sample, for the worker subgroup, and for the education/training subgroup.

**TABLE 1:** Baseline demographic, vocational/educational, diagnostic, clinical functioning, and quality of life (QoL) characteristics of the total cohort and separately for the individual placement and support (IPS) and treatment‐as‐usual (TAU) groups.

	Total sample (*N* = 94)	IPS (*N* = 48)	TAU (*N* = 46)
Gender, *n* female (%)	49 (52.1)	27 (56.3)	18 (39.1)
Age in years, mean (SD)	26.3 (4.8)	27.2 (5.0)	25.4 (4.5)
In Partnership, *n* (%)	18 (19.2)	8 (16.7)	10 (21.7)
Social relationsship/close friend, *n* (%)	76 (80.9)	42 (87.5)	34 (73.9)
Migration background, *n* (%)	41 (43.6)	19 (39.6)	22 (47.8)
Native language			
German, *n* (%)	67 (71.3)	34 (70.8)	33 (71.7)
Bilingual (German and other), *n* (%)	15 (15.9)	10 (20.8)	5 (10.9)
Other than German, *n* (%)	12 (12.8)	4 (8.4)	8 (17.4)
School‐leaving qualification			
No school‐leaving qualification, *n* (%)	2 (2.1)	1 (2.1)	1 (2.2)
2nd level, *n* (%)^a^	14 (14.9)	4 (8.3)	10 (21.7)
3rd level, *n* (%)^a^	78 (83)	43 (89.6)	35 (76.1)
Vocational training/higher education, *n* (%)	48 (51.1)	27 (56.2)	21 (45.7)
Dropout from vocational training, *n* (%)	34 (36.2)	15 (31.3)	19 (41.3)
Subsistence			
Earnings from work, *n* (%)	30 (31.9)	18 (37.5)	12 (26.1)
Social security benefits, *n* (%)	39 (41.5)	16 (33.3)	23 (50)
Educational/training loans (BaföG), *n* (%)	25 (26.6)	14 (29.2)	11 (23.9)
Under the poverty line (< 1000€ per month), *n* (%)	70 (74.5)	34 (70.8)	36 (78.3)
Debts^b^, *n* (%)	37 (39.8)	20 (42.6)	17 (37)
Amount of the debts^c^, € mean (SD)	20,358.8 (69,083.7)	27,257.9 (90,562.5)	11,620.0 (23,628.6)
Employment, education, and training status before study entry			
Competitive employment, *n* (%)	13 (13.8)	7 (14.6)	6 (13)
Education/training, *n* (%)	17 (18.1)	9 (18.8)	8 (17.4)
Not in employment. education or training (NEET), *n* (%)	64 (68.1)	32 (66.7)	32 (69.6)
Main diagnosis			
F1x.5, *n* (%)	23 (24.5)	13 (27.1)	10 (21.7)
F2x, *n* (%)	50 (63.8)	28 (58.3)	32 (69.6)
F3x, *n* (%)	11 (11.7)	7 (14.6)	4 (8.7)
Secondary diagnosis, *n* (%)	33 (35.1)	19 (39.6)	14 (30.4)
Substance missuse, *n* (%)	70 (74.5)	34 (70.8)	36 (78.3)
Treatment in (day) hospital before study entry, *n* (%)	91 (96.8)	47 (97.9)	44 (95.7)
Days since last (day) hospitalization before study entry, mean (SD)	251.2 (409.9)	265.2 (461.4)	236.3 (351.3)
PANSS^d^			
Positive, (7‐49), mean (SD)	10.7 (4.4)	10.9 (4.4)	10.5 (4.5)
Negative, (7‐49), mean (SD)	12.5 (5)	11.4 (4.2)	13.7 (5.5)
General (16‐112), mean (SD)	27.4 (8.4)	27 (8.2)	27.9 (8.6)
Total score, mean (SD)	50.7 (15.1)	49.3 (14.7)	52.2 (15.5)
GAF^e^, mean (SD)	66.6 (13.2)	67.1 (13.8)	66.1 (12.6)
ARS^f^ (1‐5), mean (SD)	4.2 (1.1)	4.3 (1)	4 (1.1)
Mini ICF‐AAP (0‐4), mean (SD)	0.8 (0.6)	0.8 (0.5)	0.9 (0.6)
QoL G‐factor^g^ (1‐10), mean (SD)	5.8 (1.7)	5.9 (1.6)	5.8 (1.8)

^a^ according to the International Standard Classification of Education (ISCED): 2nd level: 10 school years, 3rd level: 12 rsp. 13 school years.

^b^ Missings = 1. IPS = 1. TAU = 0.

^c^ Missings = 3. IPS = 1. TAU = 2.

^d^ Missings = 5. IPS = 2. TAU = 3.

^e^ Missings = 5. IPS = 2. TAU = 3.

^f^ Missings = 6. IPS = 2. TAU = 4.

^g^ Missings = 4. IPS = 3. TAU = 1.

## 2 Material and Methods

### 2.1 Study Design, Setting, and Participants

CONSORT Statement standards have been implemented (Moher et al. 2001) ( ). The study was designed as a monocenter prospective randomized single‐blind controlled parallel‐group trial. Participants were recruited between August 2017 and October 2021 from the psychiatric services at the Klinikum am Urban Berlin, Germany. The raters for assessments were blinded to the group allocation. The RCT protocol was registered at the German Clinical Trials Register (DRKS‐ID DRKS00018107).

**FIGURE 1:**
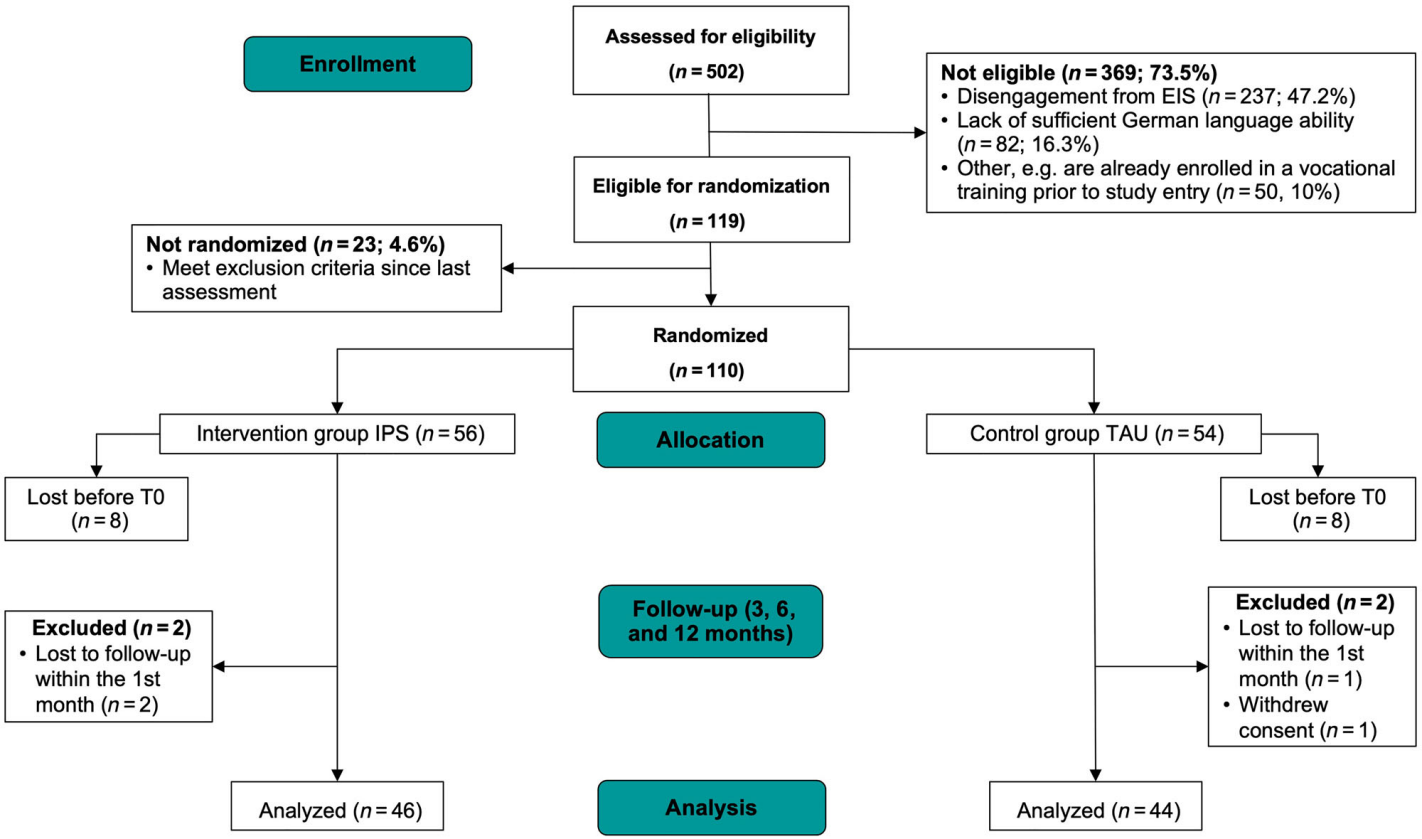
CONSORT study flowchart.

### 2.2 Participants

Young adults with EP who expressed interest in EET, independent of their education or vocational status.

### 2.3 Inclusion/Exclusion Criteria

To be included in the study, patients had to:

- be between 18 and 35 years of age;
- have a stabilized mental disorder following a clinical diagnosis of schizophrenia‐spectrum disorders (DSM‐V criteria: included schizophreniform disorder, schizophrenia, schizoaffective disorder, major depressive disorder with psychotic features, bipolar disorder with psychotic features, and psychosis NOS) within 5 years (modified after Bird et al. 2010);
- express an interest in competitive employment or professional education.

Patients exhibiting the following were excluded:

- learning disability (IQ < 70);
- without sufficient German language skills (level a1);
- primary substance abuse disorder;
- a physical or organic handicap that seriously impeded work and,
- unwillingness to attend regular outpatient treatment.

#### 2.3.1 Trial Intervention

IPS is an evidence‐based practice for helping people with SMI gain and maintain competitive employment and/or reengage in education or training with nine well‐defined key principles, which the IPS‐Y Fidelity Scale can reliably assess (Bond, Becker, et al. 2011; Bond et al. 2019). All participants in the IPS group were offered a 12‐month IPS intervention according to program fidelity. To prevent disengagement from the outpatient psychiatric treatment, the IPS specialists offered up to eight adherence therapy (AT) sessions based on the principles of motivational interviewing integrated into the IPS meetings (Staring et al. 2010; Gray et al. 2010) (Table S1).

#### 2.3.2 IPS Program Fidelity

To ensure that IPS met the fidelity standard of the IPS model, we rated its fidelity once a year by applying the “IPS‐Y: IPS fidelity scale for young adults” (Bond et al. 2019).

#### 2.3.3 Treatment as Usual

TAU took place in an outpatient psychiatric service in Berlin, Germany, a low‐threshold, interdisciplinary mental health service for young adults aged 16–35 years (McGorry et al. 2022). Individually tailored care combines evidence‐based interventions for medication, psychoeducation, psychological therapies, family intervention, social work, and general support to promote recovery and psychosocial functioning (Bechdolf et al. 2024). Social workers and counseling staff in the outpatient psychiatric services refer patients to traditional vocational rehabilitation programs.

### 2.4 Data Sources and Measures

#### 2.4.1 Data Sources

Two categories of data were examined in this study: (i) vocational/educational and (ii) nonvocational/noneducational. Data was assessed at baseline and at 3‐ and 6‐month follow‐ups. In addition, vocational/educational outcomes were assessed at the 12‐month follow‐up. Trained researchers and clinical staff assessed primary and secondary outcomes.

The primary outcome was defined as “competitive employment, education/training (EET) for at least 15 h per week.” This criterion is based on the definition of “ability to work” in the German social security system. “Ability to work” here is defined by the ability to work for at least 15 h per week/at least three hours per day. Individuals who are medically assessed and not considered able to work for at least 15 h per week/at least 3 h per day are considered “fully disabled.” Those individuals receive disability benefits and are allowed to work in sheltered workshops.

Employment was operationally defined as a competitive job on the open labor market. To be considered competitively employed, the participant had to (i) hold the job for at least 1 day and (ii) earn at least the minimum wage. Education/training is counted as mainstream education, leading to a nationally recognized vocational qualification or degree. The minimum wage in 2021 in Germany was €9.60 per hour.

#### 2.4.2 Outcome Measures

For international comparability, the variable “Employment, education, or training (EET) for at least 1 week during the 12‐month follow‐up period” was included in the secondary EET outcomes as:

- percentage participants in competitive EET;
- total weeks in EET;
- total hours in EET;
- monthly EET status during the 12‐month follow‐up period;
- total earnings in € in EET;
- Only Worker sample: (a) time to the first job (i.e., weeks from study entry to first EET start), (b) hours per week, and (c) wages per hour;
- group differences in IPS versus TAU in the education/training subgroup.

The IPS specialists were instructed to note any face‐to‐face contact, including video and phone calls, or e‐mail contact. The frequencies of the specialists’ contacts with IPS participants, supervisors, and other relevant people (such as employers, educational institutions, early intervention service staff, rehabilitation counselors, and family members/relevant others) were calculated from hospital charts and defined as IPS‐specialist performance.

At baseline, sociodemographic information was recorded, including employment/education/training, housing situation, and psychiatric treatment before the trial. The diagnosis was validated by the treating psychiatrist based on ICD‐10 criteria. Also, the following validated questionnaires were used to assess mental health status, treatment adherence, substance use, and quality of life at baseline and follow‐ups:

The Positive and Negative Syndrome Scale (PANSS) (Kay et al. 1992) was used to assess the severity of the symptoms. To determine the overall level of functioning, the Global Assessment of Functioning Scale (GAF) (Aas 2011) and the Mini‐ICF‐APP, a measure used to assess mental illness and varying degrees of disability as defined by the International Classification of Functioning (ICF) (Linden et al. 2009), were used. Adherence and quality of life were measured with the Adherence Rating Scale (ARS) (Staring et al. 2010) and the Modular System for Quality of Life (MSLQ‐R) (Pukrop et al. 2000), respectively. Additional instruments are listed in Supporting Information S2.

#### 2.4.3 Data Management and Analysis

The primary outcome was defined in accordance with Fowler (Fowler et al. 2009): the number of patients in EET (at least 15 h per week) that would lead to a nationally recognized vocational qualification or degree in the 12‐month follow‐up period.

First, intention‐to‐treat analysis (ITT) was performed. Then, outcome and subgroup analysis were performed. Independent t‐tests were used to compare the difference between group means. For longitudinal data, repeated‐measures analysis of variance (ANOVA) under the general linear model was used to examine the effects of time, group, and time‐by‐group interaction.

Data proportions of categorical variables using *χ* ^2^‐tests with odds ratios were performed. Values of continuous variables between the two groups were compared using either the *t*‐test or, for non‐normally distributed variables, the Wilcoxon ranks sum test. A random effects logistic regression was used to assess overall differences between the IPS‐AT and TAU groups in month‐by‐month EET rates for the 12‐month follow‐up. Pearson correlations were used to test the linear relationship between the intensity of IPS support and the duration of employment and/or education/training. Statistical tests were two‐sided and performed with a 5% significance level, reporting a 95% CI and the *p* value of the effect. Data was analyzed using JMP 17.2 software (SAS Institute, Cary, NC, USA) and the Statistical Package for the Social Sciences 24.0 (SPSS, IBM Analytics, Armonk, New York).

#### 2.4.4 Ethical Considerations

The study was reviewed and approved by the Ethics Committee of the Berlin Medical Association Eth‐13/17, date of the vote: 2017‐07‐05. The participants provided written informed consent accompanied by trial information before study inclusion, and the ethical principles of the Helsinki Declaration were followed.

## 3 Results

A total of 110 patients were randomized; 16 were lost before T0. Regarding the vocational and educational outcome data, 90 of 94 (95.7%) participants were available Figure 1.

### 3.1 Sample Description

Baseline data included clinical variables, demographic, vocational, and educational participant characteristics1.

Participants with completed secondary education were twice as likely to be in EET at baseline compared to those without: 20 participants (41.2%) versus 9 (19.6%), respectively (*χ* ^2^(1) = 5.38, *p* = 0.02). Of the participants aged 25 years or younger, 39 of 51 (76.5%) had not yet completed secondary education at the study baseline.

### 3.2 Primary EET Outcomes

The percentage of participants in EET for at least 15 h per week for at least 1 week in the 12‐month follow‐up period was significantly higher in the IPS group, 78.3% (36/46), compared to 54.6% (24/44) in the TAU group (*χ* ^2^(1) = 5.69, *p* = 0.02, OR = 3.0, CI = 1.2–7.51).

Adjusted for the “NEET‐status” at baseline, the EET rate decreased for both groups: 71% (22/31) in the IPS group and 40% (12/30) in the control group at the 12‐month follow‐up, *χ* ^2^(1) = 5.93, *p* = 0.015, OR = 3.67, CI = 1.26–10.64. The odds of being in EET for at least 15 h per week were 3.67 times higher in IPS compared to TAU. The analysis of the employment subgroup, participants who were not in education or training during the follow‐up period, reveals *χ* ^2^(1) = 4.47, *p* = 0.03, OR = 3.5, CI = 1.07–11.4.

### 3.3 Secondary EET Outcomes

The percentage of EET and its sustainability, defined as “at least 50% of the 12‐month follow‐up in EET,” are significantly higher for the IPS than for the controls. The EET duration in hours and the weekly time in EET are also significantly greater for the IPS group than for the control group. Finally, the total wages were almost twice as high for the participants in the IPS than for the TAU group.

Table 2 summarizes the results of EET in the IPS and the control group.

**TABLE 2:** Secondary outcomes in employment, education/training (EET).

	IPS (*N* = 46)	TAU (*N* = 44)	*p*	Effect size OR, Cohen's *d*	CI 95%
EET at least one day, *n* (%)	38 (82.6)	26 (59.1)	0.02	3.29	1.25–8.68
EET sustainability, *n* (%)	30 (65.2)	15 (34.1)	< 0.01	3.63	1.52–8.65
EET in weeks, mean (SD)	31.5 (20.5)	18.37 (21.2)	< 0.01	0.63	0.21–1.1
EET in hours, mean (SD)	1025.96 (790.94)	565.55 (707.11)	< 0.01	0.61	0.19–1.03
Earnings in €, mean (SD)	10,242.33 (13,436.9)	5216.56 (7871.4)	0.03	0.45	0.04–0.87

**FIGURE 2:**
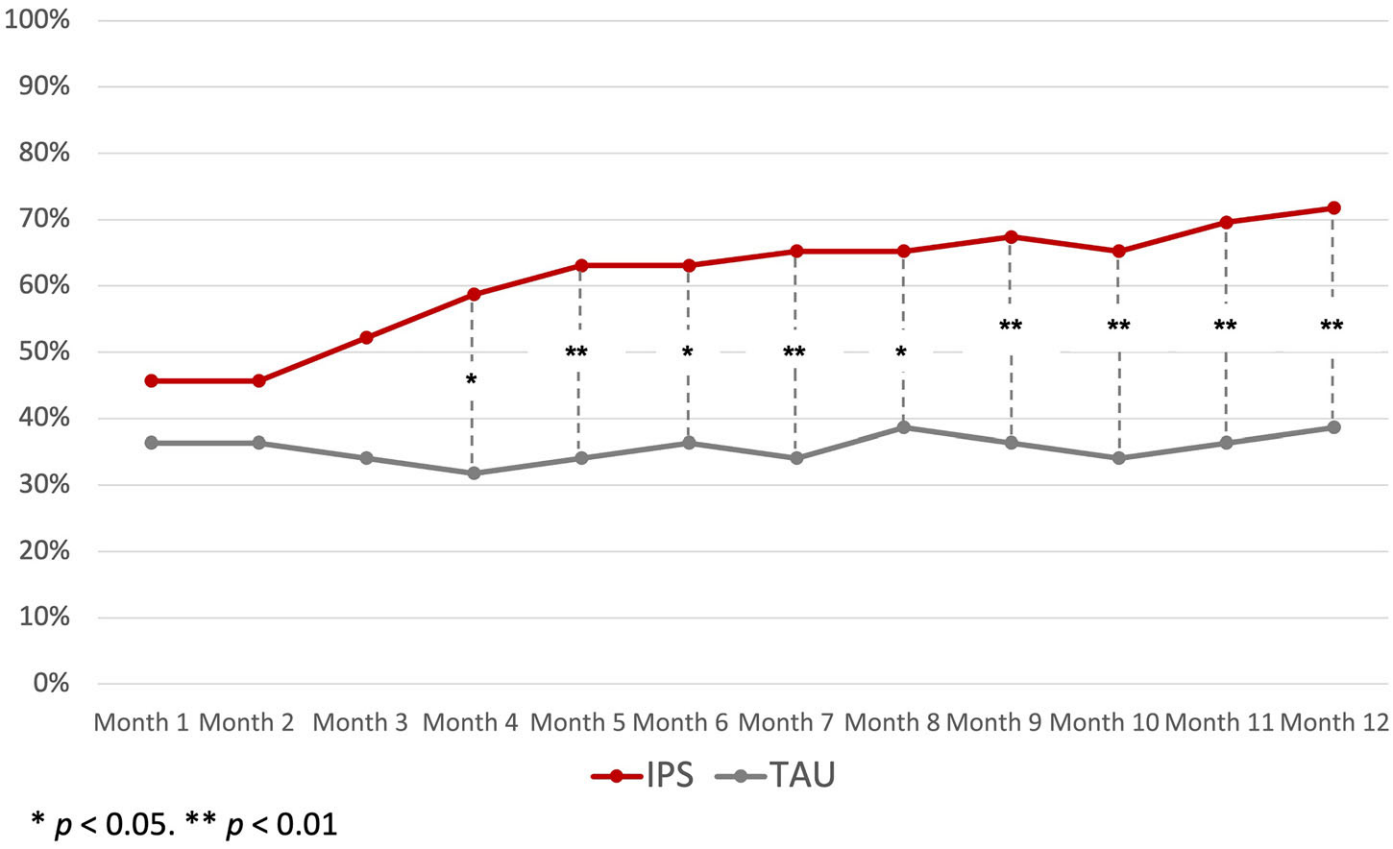
Monthly EET rates during the 12‐month follow‐up period.

The monthly rates of EET for each group are graphed in Figure 2. Starting in Month 4, the IPS group showed consistently higher EET rates according to univariate tests of proportions.

We found a significant group effect, *F*(1, 88) = 8.94, *p *< 0.01; a significant time effect, *F*(11, 968) = 2.52, *p *< 0.004; and a significant group × time interaction, *F*(11, 968) = 2.09, *p* = 0.02. This analysis substantiates the graphical pattern of a higher monthly EET rate over the follow‐up period, explicitly favoring IPS over the control group from Month 4 onwards.

Throughout the 12‐month follow‐up period, participants in the IPS group (*N* = 46) had an average of 17.17 (SD = 9.82, range 3–43) IPS‐specialist contacts. The mean frequency of IPS‐specialist contact was positively correlated with total weeks in EET over the follow‐up period (*r* = 0.12, *p* = 0.0). The sustainability of EET was also correlated with the intensity of contact with the IPS specialists: a higher frequency of IPS contacts correlated significantly with higher EET rates (*N* = 30), *M* = 20.03, SD = 9.27 versus (*N* = 16), *M* = 11.81, SD = 8.73, *t*(44) = 2.92, *p *< .01.

In the subsample of the competitively employed participants (*N* = 36), no significant differences were found between the IPS and the TAU group regarding the time to start the first job in weeks *M* = 9.3, SD = 12.5 versus *M* = 10.9, SD = 14.5. Likewise, no group differences were found in weekly working hours *M* = 33.24, SD = 10.2 versus *M* = 29.60, SD = 12.6, and hourly wages € *M* = 12.9, SD = 6.5 versus *M* = 10.5, SD = 3.3.

### 3.4 Educational Outcomes

Excluding all participants who never received education or training during the follow‐up period, we compared the two subsamples IPS (*N* = 25) versus TAU (*N* = 29) in education/training.

Table 3 summarizes the results of ET of the IPS and the TAU subsample.

**TABLE 3:** Subsample education/training (ET) secondary educational outcomes.

	**IPS (*N* = 25)**	**TAU (*N* = 29)**	***p***	**Effect size OR, Cohen's *d***	**CI 95%**
Education/training at least one day, *n* (%)	17 (68.0)	11 (37.9)	0.03	3.48	1.13–10.73
Education/training sustainability, *n* (%)	14 (56.0)	9 (31.0)	0.06	2.83	0.93–8.62
Duration of education/training in weeks, mean (SD)	27.69 (22.6)	14.38 (21.7)	0.03	0.63	0.06–1.15
Duration of education/training in hours, mean (SD)	867.04 (829.6)	414.72 (628.83)	0.03	0.62	0.07–1.17

A total of 59.3% (32/54) of the participants in the whole education subsample did not have completed postsecondary education at study entry. In the IPS‐education group, 78.6% (11/14) succeeded in continuing or resuming education/training compared to 33.3% (6/18) of the participants in the TAU‐education group, *χ* ^2^ (1) = 6.47, *p* = 0.01, OR = 7.3, CI = 1.47–36.66. Of the participants in the IPS‐education group, 4% (1/25) finished higher education, whereas, in the education/training control group, no one completed a training or higher education degree *χ* ^2^ (1) = 1.18, *p* = 0.28,

### 3.5 IPS Fidelity

At the beginning of the study, August 2017, IPS‐fidelity scored 104 (good fidelity) out of 125 points and remained at this level until April 2020. Fidelity was then lowered to 88 points (moderate level) and increased to 103 points in March of 2021, remaining until the study's end in October 2021.

## 4 Discussion

To our knowledge, this is the first study in the German healthcare system assessing the efficacy of IPS on employment, education, and training in young adults with EP. Over a third of the study participants had dropped out of at least one education/training program before study entry, and almost half had not completed postsecondary education/training. 69% of the study participants were NEET at baseline, which may put the study group at risk of not entering the workforce.

We found a significant difference of nearly 24% in EET between the IPS and TAU groups. After controlling for the NEET status at baseline, the difference increased to 31%. According to our hypothesis, the results of the 12‐month RCT demonstrate the superiority of IPS relating to the primary outcome rates “employment, education/training (EET) for at least 15 h per week” compared to TAU (78% vs. 54%). Beyond that, the study findings indicate that IPS is superior in the widely used outcome definition “employment, education/training at some time in the 12‐month follow‐up period,” the duration of EET, and the wages. The employment rate of our study was 30% greater for IPS than for the control group. This result aligns with the employment rate at a follow‐up of at least 20% in favor of IPS Bond et al. (2023), summarized in their meta‐analysis.

Regarding the outcomes of the worker‐only subsample, we did not find significant differences in weekly working hours, hourly wages, or time to first job. The lack of differences is likely due to the small size of this subsample, consisting of only 36 participants, which does not provide enough power to detect any effect differences. The time taken for this subgroup to find a new job was 9.3 weeks for the IPS group and 10.9 weeks for the TAU group, which is similar to the study results reported by Bond et al. (2024).

The results indicate that the effects of IPS are ongoing in the long term. After 12 months, 71% of the IPS group was still in EET compared to 39% of the control group. As a result, the participants of the IPS group had a greater percentage of sustained EET, as seen by a significantly longer tenure of EET, with 32 versus 18 weeks in the 12‐month follow‐up. In line with the findings of Erickson et al. (2020), the IPS participants reached EET after around 3 months, but not right from the beginning. In sum, in our study, IPS was superior in all vocational and educational outcomes compared to the controls (Bond et al. 2023; Bond et al. 2024; Nischk et al. 2023).

Regarding the outcomes of the subsample in education/training (Bond et al. 2024), we found favorable differences in the outcome criterion “at some time in education/training” for the IPS group. The differences between the two groups found in the measure of sustainability of actively attending educational/training programs only had medium‐sized statistical significance. The primary cause of this may be attributed to statistical power, as we calculated the sample size for the combined outcome “employment, education/training (EET) for at least 15 h per week” and not for the single entities of employment or education/training.

At the baseline of our study, only a few participants were in competitive employment (IPS 15.2% vs. TAU 13.6%) or in education/training (IPS 17.4% vs. TAU 18.2%), which is similar to the samples of Erickson et al. (2020) and Killackey et al. (2019).

All participants in the IPS group in competitive employment at baseline remained employed, and those in education/training remained at the end of the study. One participant completed higher education and took up a job with the acquired qualification during the study period. In contrast, a third of the participants in the TAU group dropped out of their jobs or education/training and were disengaged from EET at the 12‐month follow‐up. These findings demonstrate the additional value of IPS in enhancing the sustainability of postsecondary education attainment as a strong facilitator of a successful transition from youth to adulthood and later competitive employment. The potential of IPS in preventing young adults with mental health issues from being excluded from working life has positive effects on mental health. Thus, IPS should be given high attention in the treatment of young adults with EP (Gariépy et al. 2022; Maraj et al. 2019; Lindhardt et al. 2022; Erickson et al. 2020; Cotton et al. 2019), and they should receive rapid support if they stumble in their education or in their jobs (Sveinsdottir et al. 2019; Fioritti et al. 2024; Sabella 2021).

All study participants received the standard, evidence‐based treatment in the outpatient psychiatric services. The control group did not improve their vocational or educational functionality regarding employment, education, or training. These findings align with those of the meta‐analysis by Bond et al. (2015).

We found a statistical relationship between the number of IPS‐specialist performances and the duration of maintaining EET in weeks and hours. These results resemble the findings of Bond and Kukla (2011), who found a positive correlation between the frequency of contacts and months in employment at the 12‐month follow‐up. There is evidence that EET rates decline when support ends, and, in turn, the outcome can be maintained if IPS is offered as long‐term support based on individual needs (Killackey et al. 2019; Kawohl et al. 2015; Pichler et al. 2021). Therefore, the sustainability of competitive employment and the completion of mainstream education that leads to a vocational qualification or degree may only be achieved through the implementation of long‐term support.

Surprisingly, the study participants’ adherence at baseline was as high as, or even higher than, after the treatment AT in reference studies (Chien et al. 2024 Chien et al. 2016). The reason for this could be that psychoeducation is a mandatory part of inpatient psychiatric treatment. There could also be a selection bias in that young adults with EP who are interested in EET have higher adherence than those who are not interested in EET.

In conclusion, our study results show that IPS has significant advantages in assisting young adults with EP to resume employment and/or education/training and to increase their involvement in these activities over time. Combined with the results of other RCTs assessing IPS for young adults with mental health conditions (Bond et al. 2023; Al‐Abdulmunem et al. 2023; Christensen et al. 2021), IPS should be offered to young adults in outpatient psychiatric services as standard treatment according to IPS‐Y fidelity to find and maintain EET (Jäckel et al. 2023).

## Author Contributions

Conceptualization: Leopold, Jäckel Data curation: Gamig Formal analysis: Jäckel Funding acquisition: Leopold Investigation/Conducting a research and investigation process: Gamig, Mößnang, Kallenbach Methodology: Jäckel, Leopold, Bechdolf Project administration: Jäckel, Leopold Resources: Bechdolf Software: Jäckel Supervision: Leopold, Jäckel, Bechdolf, Burkhardt Validation: Leopold Visualization: Jäckel Writing–original draft: Jäckel Writing–review & editing: Jäckel, Leopold.

## Conflicts of Interest

Dorothea Jäckel has been a consultant and/or advisor to or has received honoraria and travel support from Lundbeck, Otsuka (outside the submitted work), Lilly, and Ferring. Andreas Bechdolf has been an advisor and received speakers’ honoraria and travel support from Janssen‐Cilag, Lundbeck, Otsuka, and Recordati. He also received grant support from Janssen‐Cilag and Otsuka. Karolina Leopold has been a consultant and/or advisor to or has received honoraria and travel support from Boehringer Ingelheim, Janssen/J&J, Lundbeck, Otsuka, Recordati, and ROVI. She has received grant support from Janssen/J&J and Otsuka. All mentioned conflicts of interest are unrelated to the present article. Other authors declare no conflicts of interest.

### Peer Review

The peer review history for this article is available at https://publons.com/publon/10.1002/brb3.70469.

## Supporting information



Supporting Information

## Data Availability

Data is available on request due to privacy/ethical restrictions.
