# Supplementary material for: Efficacy of Individual Placement and Support (IPS) on Employment, Education, and Training in Young Adults With Early Psychosis—A Randomized Controlled Trial
Source: Brain Behav. 2025 May 5;15(5):e70469. doi: 10.1002/brb3.70469 (PMC12053063; doi:10.1002/brb3.70469)
Supplement: Supplementary file 1 — Supporting Information [file BRB3-15-e70469-s001.docx]

Supplement 1: Content Adherence therapy

| 0) Adherence Assesment  An individual-centered approach to evaluating adherence to prescribed medical treatment plans using a structured self-report, including:   - Practical considerations - Side effects, problems, and concerns - Individual importance, beliefs, priorities, self-confidence, and satisfaction | |
| --- | --- |
| Five key elements of adherence: | |
| 1) Problem Solving | - Problem-Identification, discussing significant or potentially serious issues - Goal-setting - Evaluation of potential solutions - Creating an action plan |
| 2) Looking back | - Exploring previous treatment experiences through a detailed review of medical history on a timeline - Marking significant illness phases, life events, social and therapeutic support - Identifying helpful resources |
| 3) Exploring and resolving ambivalence | - Helping patients make an informed decision about medication adherence, using a pro and contra list |
| 4) Talking about beliefs and concerns | - Exploring beliefs that may influence patients decisions about taking medication - Rating the accuracy of beliefs on a percentage scale (0% = not accurate at all; 100% = extremely accurate) - Exploring reasons for their conviction |
| 5) Looking forward | - Helping patients understand the long-term need for medication to achieve self-identified goals - Addressing goals and potential barriers |

Supplement 2:

| Visit | t0 | t1 | t2 |
| --- | --- | --- | --- |
| time point | Baseline | after 13 weeks | after 26 weeks |
| Sociodemographic, medical treatmentdata | X |  |  |
| Medical Treatment | X | X | X |
| Insight and Treatment Attitude Questionnaire (ITAQ)^1^ | X | X | X |
| Drug Attitude Inventory (DAI)^2^ | X | X | X |
| Addiction Severity Index^3^ | X | X | X |
| Side effects UKU^4^ | X | X | X |
| Hospitalization and mental health service use | X | X | X |

*dose, application, long-acting injectable antipsychotics and substance of psychopharmcotherapy

Measures of Drug Attitudes (DAI) [41, 42], Substance consumption and Addiction Severity (ASI) [43], psycho pharmacotherapy (daily dose, application, and substance), and side effects by using the UKU [44], hospitalizations, and mental health service use assessed by the medical records will be reported elsewhere analyzing moderators, mediators and confounder of treatment effects at 6- and 12-months follow-up.

^1)^McEvoy JP; Apperson LJ; Appelbaum PS; Ortlip P; Brecosky J; Hammill K; Geller JL; Roth L: Insight and Treatment Attitudes Questionnaire (ITAQ). Journal of Nervous & Mental Disease, 1989. 177(1): p. 43-47

^2)^Townsend, L., J. Floersch, and R.L. Findling, *Adolescent attitudes toward psychiatric medication: The utility of the Drug Attitude Inventory.* Journal of Child Psychology and Psychiatry, 2009. **50**(12): p. 1523-1531.

^3)^McLellan, A.T., D. Carise, and T.H. Coyne, *Addiction Severity Index 5th Edition.* J Subst Abuse Treat, 1992. **9**: p. 199-213.

^4)^Lingjærde, O., et al., *The UKU side effect rating scale: A new comprehensive rating scale for psychotropic drugs and a cross-sectional study of side effects in neuroleptic-treated patients.* Acta Psychiatrica Scandinavica, 1987. **76**(Suppl 334): p. 100.
